# Designing siRNA/chitosan-methacrylate complex nanolipogel for prolonged gene silencing effects

**DOI:** 10.1038/s41598-022-07554-0

**Published:** 2022-03-03

**Authors:** Ye Cao, Yang Fei Tan, Yee Shan Wong, Muhammad Aminuddin, Bhuthalingam Ramya, Melvin Wen Jie Liew, Jiaxin Liu, Subbu S. Venkatraman

**Affiliations:** 1 Institute of Blood Transfusion, Chinese Academy of Medical Sciences and Peking Union Medical College, Chengdu, China; 2 School of Materials Science and Engineering, National University of Singapore, Singapore, Singapore; 3 School of Materials Science and Engineering, Nanyang Technological University, Singapore, Singapore

**Keywords:** Drug discovery, Nanoscience and technology

## Abstract

Despite immense revolutionary therapeutics potential, sustaining release of active small interfering RNA (siRNA) remains an arduous challenge. The development of nanoparticles with siRNA sustained release capabilities provides an avenue to enhance the therapeutic efficacy of gene-based therapy. Herein, we present a new system based on the encapsulation of siRNA/chitosan-methacrylate (CMA) complexes into liposomes to form UV crosslinkable Nanolipogels (NLGs) with sustained siRNA-release properties in vitro. We demonstrated that the CMA nanogel in NLGs can enhance the encapsulation efficiency of siRNA and provide sustained release of siRNA up to 28 days. To understand the particle mechanism of cellular entry, multiple endocytic inhibitors have been used to investigate its endocytosis pathways. The study saw positively charged NLGs entering cells via multiple endocytosis pathways, facilitating endosomal escape and slowly releasing siRNA into the cytoplasm. Transfection experiments confirmed that the crosslinked NLG delivery system provides effective transfection and prolonged silencing effect up to 14 days in cell cultures. We expect that this sustained-release siRNA NLG platform would be of interest in both fundamental biological studies and in clinical applications to extend the use of siRNA-based therapies.

## Introduction

Small interfering RNA (siRNA) emerged in the 1990s as a powerful therapeutic approach for gene silencing^1–3^. However, success in clinical trials has been limited owing to the extracellular and intracellular barriers, such as siRNA stability issue, difficulty in entering cells and intracellular trafficking. Various approaches are investigated to overcome the barriers via the use of delivery systems^4,5^, such as solid lipid nanoparticles^6^, complexation with cationic carriers or molecules^7^, fusogenic peptides^8^, layer by layer nanoparticle^9^ and positively charged lipids^10^. However, it is noteworthy that the majority of reported siRNA nano delivery systems, including those under clinical trials, do not have sustained-release capabilities, and their therapeutic efficacy may be limited by the duration of gene silencing action.

Lipid based nanoparticles have been extensively explored as nonviral based gene delivery carriers and represented one of the most promising delivery systems for siRNA. For instance, lipoplex, formed by complexation of negatively charged siRNA with cationic lipids, is one of the most widely studied non-viral delivery systems^11,12^. However, most lipoplex formulations present toxicity issues. Furthermore, the equilibrium structures and actual transfection mechanisms of lipoplexes are unclear^13^. A few cationic lipid/polymer-based nanosystems, such as Lipofectamine and polyplexes, have been successfully developed and moved into clinical trials^3,6^, but none of them have been approved. Recently, lipid nanoparticles (LNPs) have garnered increasing attention in this field because FDA approved Onpattro in 2018 for targeting polyneuropathy caused by amyloidosis. Onpattro nanoparticles are LNP-siRNA complex cores, formed by electrostatic interaction between ionizable lipids (positive charge when pH < 7) and siRNAs, which are in turn surrounded by a layer of lipids, cholesterol and PEG conjugated lipids. Similarly, SARS-CoV-2 vaccines from Moderna and BioNTech/Pfizer are also using LNP system to deliver mRNAs^14^. After LNPs enter cells, their ionizable lipids become positively charged, facilitating the LNPs’ escape from endosomes, enabling their cargo release into the cytosol. The LNPs’ ionizable lipids lose their positive charges in the neutrally-charged cytosol resulting in the burst release of the complexed negatively charged nucleic acids into the cytosol (without sustained release behavior). Indeed, none of the above mentioned systems have controlled release ability and thus have to be administered repeatedly to achieve therapeutic effect for a prolonged period. Frequent administration of therapeutic gene drugs required for extended therapeutic effect is not patient-friendly and may often lead to undesirable systemic side effects^15^.

Inspired by the LNPs, we propose a core/shell structure chitosan (CS) -based nanolipogel (NLG) system to control release of siRNA. Similar to the LNPs, chitosan-methacrylate (CMA) form nano-complexes with siRNA as cores. These cores are then in turn encapsulated into liposomes to form UV crosslinkable NLGs to sustain release siRNA. Chitosan is a well-known cationic polysaccharide that has been widely explored for gene delivery including siRNA^16,17^. However, their usage in terms of physicochemical and biological properties can be massively hindered for sustained delivery in vivo as chitosan (pKa = 5.6) can lose its positive charge at pH 7.4, rapidly releasing all the cargo from CS nanoparticles^18^, either by uncrosslinking or by the debonding of the bound cargo. To prevent or minimize this rapid release, lipid bilayers have been coated on the surface of the prepared CS nanoparticles^19^. Currently, the majority of the CS-lipid hybrid nanoparticles are fabricated by ionic gelation between chitosan and sodium tripolyphosphate (TPP) followed by coating with lipid bilayers^19–21^. However, these core–shell structured CS nanoparticles still suffer from particle size uniformity issue, incomplete lipid coating, complicated preparation process and rapid drug release^19^. For instance, hyaluronic-acid modified lipid-coated CS nanoparticles were explored for the delivery of moxifloxacin hydrochloride, with a crosslinked core of chitosan and TPP incorporating the drug, followed by lipid coating. The polydispersity index (PDI) of the particles were in a broad range from 0.24 to 0.39, and the nanoparticles released up to 60% of the drug within 180 min of release study^21^. In another example, Min Jiang et al. reported cationic lipid-coated TPP crosslinked chitosan nanoparticles for the delivery of plasmids. Their nanoparticle only exhibited gene transfection for a limited period (2 days)^20^. Evidently, it is important to design carriers with the abilities to escape the endosomes following endocytic entry and, in the case of siRNA-based gene silencing, slowly release the loaded siRNA into the cytoplasm.

**Figure 1:**
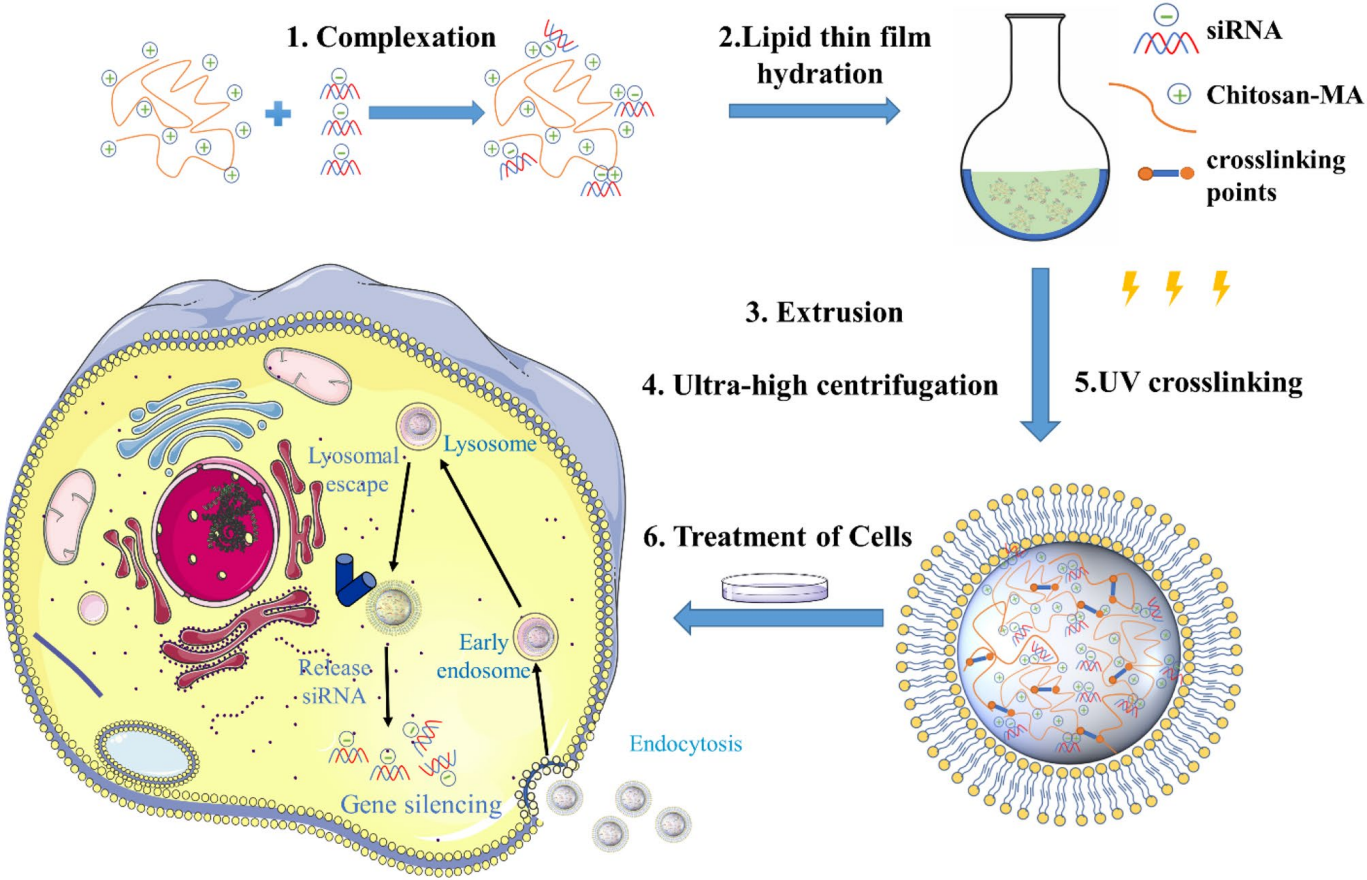
Fabrication of UV crosslinked chitosan-methacrylate based Nanolipogel. CMA and siRNA were mixed first and then used to hydrate lipid thin film. The formed MLV were extruded and downsized to LUV. After purification by ultra-high centrifugation, NLGs were achieved after UV crosslinking. NLGs were treated with the cells to study the gene silencing effect. The cell structure image was modified from original artwork by Servier Medical Art (https://smart.servier.com/).

Special design modifications should be taken into consideration to prevent premature efflux of the siRNA before and after cellular entry. Herein, we designated covalently crosslinked nanogel core in NLGs to control the release of siRNA, while lipid bilayers were designed to protect the chitosan-siRNA nanocomplex from dissociation at physiological pH and further facilitate the cellular internalization and intracellular trafficking. To start with, a lipid film is formed by the conventional process and then hydrated with the crosslinkable aqueous mixture of chitosan-methacrylate (CMA) polymer and siRNA (Fig. 1). The synthesized CMA can readily form a complex with siRNA via electrostatic interaction, thus offering enhanced encapsulation efficiency and some protection against degradation by nucleases, and then be further crosslinked to increase the structural stability of the nanostructure. Experiments inclusive of drug release, cellular entry, endocytotic pathway, toxicity, intracellular trafficking and gene silencing activity of siRNA encapsulated in NLGs using human foreskin fibroblast were performed to validate our concept of a nanocarrier incorporating siRNA that can enter cells, escape the endosome and then slowly release the siRNA into the cytosol of cells over a period.

## Results

### Characterization of CMA and NLGs

**Figure 2:**
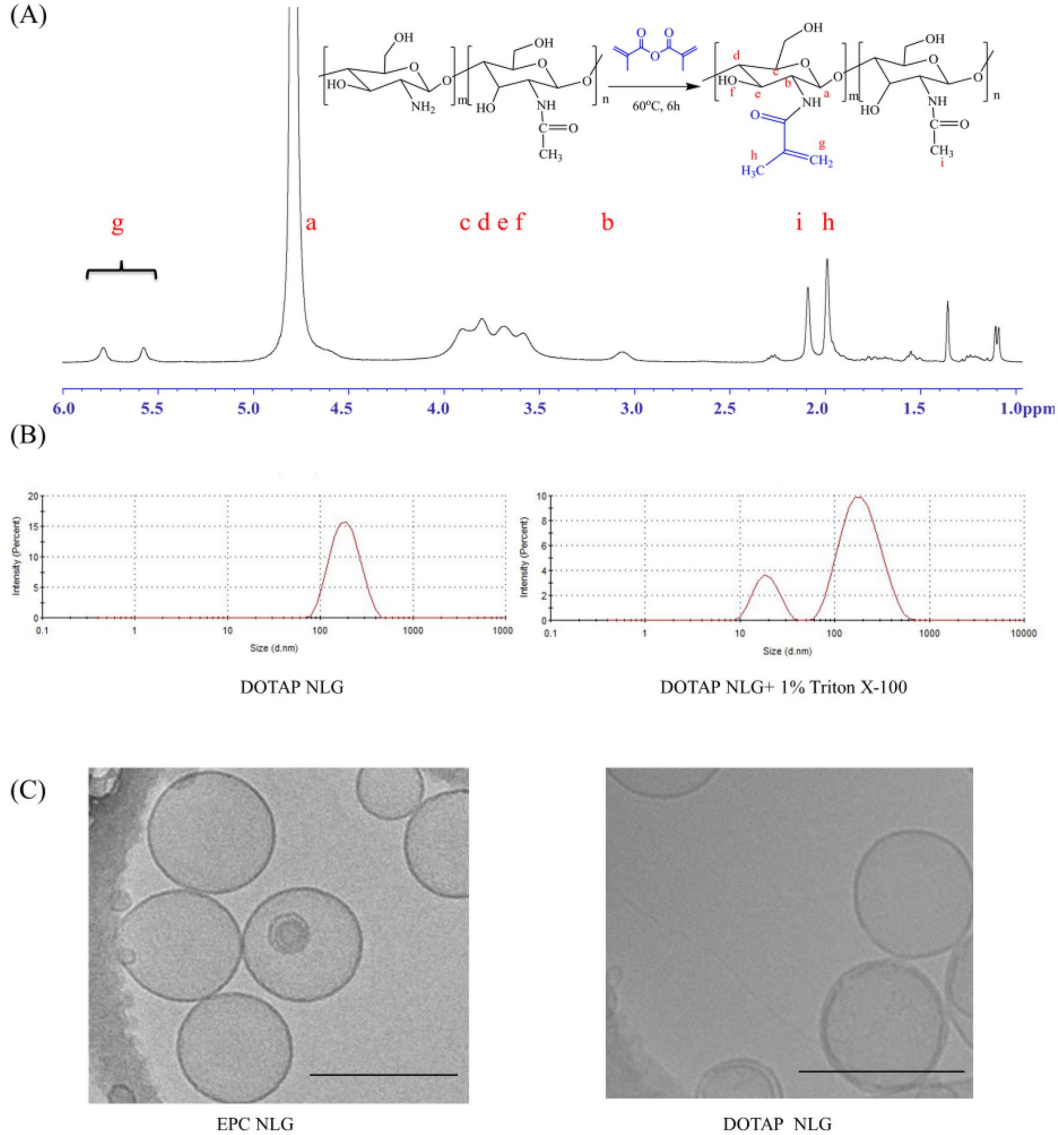
(**A**) NMR of chitosan-methacrylate; (**B**) DLS size results of DOTAP NLG and DOTAP NLG + 1% Triton X-100; (**C**) Cryo-TEM images of crosslinked EPC NLG and DOTAP NLGs. (Scale bar: 200 nm).

Chitosan is a natural polymer containing various primary amino groups, which can be modified according to the application. It is well known that chitosan is insoluble in neutral or alkaline solution because of the presence of numerous hydrogen bonds. To enhance water solubility of chitosan and better control release of siRNA, we proposed the use of CMA via conjugating methacrylate groups to the chitosan chains. The modification of methacryloyl groups to chitosan serves dual roles in making it hydro-soluble and UV crosslinkable. CMA was synthesized by conjugating methacryloyl groups to amino groups of CS chains. The chemical structure of CMA was characterized by ^1^H NMR spectrum (Fig. 2A), which showed the existence of vinyl protons at 5.57 and 5.78 ppm (g, 2H, CH_2_), methyl protons of methacrylic anhydride residues at 1.88 ppm (h, 3H, CH_3_). Literature supported that there is no chemical shift showed at 5.5–6.0 ppm in native chitosan ^1^H NMR spectrum; and only vinyl proton has the chemical shift peaks at 5.5–6.0 ppm^22,23^. SD% was a key parameter to determine the modification degree of amino groups in chitosan. The SD% of CMA was about 21.9% calculated from the ^1^H NMR spectrum using Eq. (1). Therefore, ^1^H NMR spectra result confirmed the successful conjugation between chitosan’s amino groups and methacrylic anhydride. In addition, the Mw and Mn of CMA measured by SEC were 17,272 and 9800, respectively.

Table 1 illustrated the DLS size and zeta potential data of NLGs and NLPs. All the NLGs and NLPs have similar hydrodynamic diameters of ~ 150 nm and PDIs < 0.2, which demonstrated that this liposomal template method could fabricate uniform nanoparticles. Figure 2B exhibited the typical DLS size plot of DOTAP NLG. After stripping off lipid bilayers by Triton X-100, the right figure in Fig. 2B showed two peaks at 10 and 100 nm, representing the micelle and CS core formation, respectively. The zeta potential of CMA and CMA/siRNA complex was 36.87 ± 1.39 and 17.1 ± 0.7 mV, respectively. After the encapsulation of the CMA/siRNA complex in EPC NLGs, the zeta potential of EPC NLGs and NLPs was 1.5 ± 2.2 and -5.2 ± 2.4 mV. Thus, the surface charge of EPC NLGs and NLPs were neutral. The surface charge of DOTAP NLGs was 42.3 ± 1.2 mV, while that of DOTAP NLPs was 27.4 ± 0.9 mV. The charge of DOTAP NLPs was lower than that of DOTAP NLGs because siRNA might form complex with DOTAP lipid during the hydration and thus neutralize the charge of DOTAP. In terms of drug encapsulation, the EE of NLGs is significantly higher than that of NLPs due to the complex formation between CMA and siRNA. DOTAP NLGs has lower EE compared to EPC NLGs possibly due to the repulsive force between complex and DOTAP. Further, the morphology was characterized by Cryo-TEM. As seen in Fig. 2C, spherical nanostructures and lipid bilayer were observed for both NLGs and the particle size shown ranged from 100 to 200 nm, which is consistent with the size data by dynamic light scattering.

**Table 1:** CMA nanolipogel and bare nanoliposomes characterization.

Sample	Nanoliposomes (NLP)		Nanolipogels (NLG)	
	EPC NLP	DOTAP NLP	EPC NLG	DOTAP NLG
Size (d, nm)	147.6 ± 7.6	151.1 ± 2.1	163.6 ± 8.8	154.4 ± 6.5
PDI	0.16 ± 0.01	0.23 ± 0.01	0.12 ± 0.07	0.15 ± 0.03
Zeta potential (mV)	− 5.2 ± 2.4	27.4 ± 0.9	1.5 ± 2.2	42.3 ± 1.2
EE (%)	16.9 ± 1.2	20.7 ± 2.3	86.9 ± 1.2	79.1 ± 1.3

### In vitro release study

**Figure 3:**
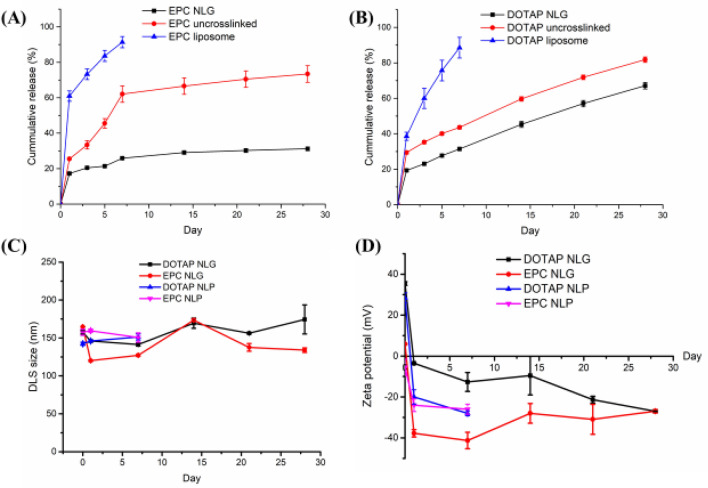
Sustained-release profiles of siRNA encapsulating in (**A**) EPC NLPs, uncrosslinked and crosslinked EPC NLGs, (**B**) DOTAP NLPs, uncrosslinked and crosslinked DOTAP NLGs, (**C**) Size change during release, and (**D**) Zeta potential change during release (n = 3).

For release kinetic studies, the siRNA release behavior from NLPs, uncrosslinked and crosslinked chitosan NLGs, were monitored for 28 days under physiologically relevant in vitro conditions (pH 7.4, 37 °C) as shown in Fig. 3. Both EPC and DOTAP NLPs showed a large initial burst release for the first 24 h. Approximately 60% and 40% of siRNA were released from EPC NLP and DOTAP NLP, respectively and release was completed on day 7.

Compared to NLPs that display a huge burst release of siRNA, NLGs that contain the additional chitosan nanogel core (both un-crosslinked and crosslinked) in their structure, can significantly suppress the initial burst and prolong the release time. As for the EPC based nanosystems (Fig. 3A), the initial burst release of siRNA from uncrosslinked EPC NLG was reduced to 25% on the first day, with an average time to 50% release (“half-life of release”) of about 5 days. Once the CMA was crosslinked, the crosslinked NLGs further slowdown the release, resulting in a reduction in the initial burst to about 18% and a sustained release of 32% up to day 28.

In contrast, for the DOTAP NLG formulations (both uncrosslinked and crosslinked), the siRNA release profiles had a 24 h initial burst release period that was followed by a gradual, approximately linear release until day 28 (Fig. 3B). The linear release was probably due to strong ionic interactions between siRNA and chitosan. By day 28, the extent of siRNA release for the un-crosslinked and crosslinked DOTAP NLGs was approximately 80% and 65%, respectively. Furthermore, the size and zeta potential change of NLGs during the release study was monitored (Fig. 3C,D). Both EPC and DOTAP NLGs were stable in size up to 28 days. At day 0, zeta potential of both DOTAP NLG and DOTAP NLP was positively charged and then dropped rapidly after one day of release. The initial zeta potential drop was due to the release of highly negatively charged siRNA from the NLGs and zeta potential maintained stable for the sequent release period. Zeta potential of EPC NLGs and EPC NLPs were neutral charged at day 0 and then dropped rapidly due to the release of siRNA.

### In vitro cytotoxicity and cellular entry

**Figure 4:**
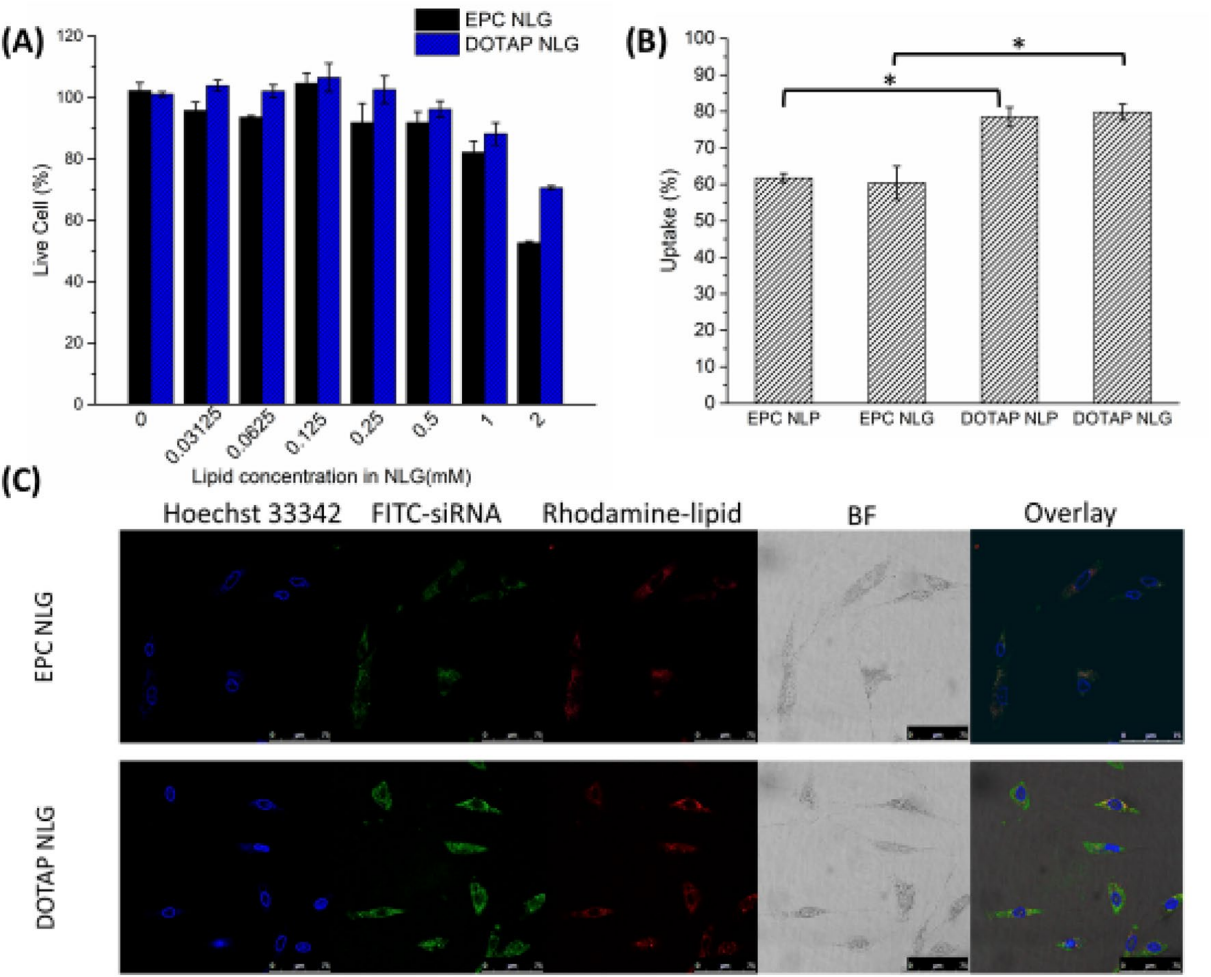
NLGs regulates in vitro cellular uptake. (**A**) Relative cytotoxicity and (**B**) cellular uptake of engineered NLPs and NLGs. (*:*p* < 0.05) (**C**) Fluorescent microscope images of fibroGRO cellular uptake of synthesized NLPs and NLGs. Blue: Hoechst 33,342, Green: FITC-siRNAs, Red: Rhodamine-labeled lipid, BF: bright field. The scale bar represents 75 µm.

Results concerning the toxicity of various NLG systems are shown in Fig. 4A. No significant cytotoxicity was observed for both EPC and DOTAP NLGs at the tested lipid concentrations between 0.03125 and 0.5 mM, but a slight reduction in cell viability was observed above 1 mM. This observation was consistent with Alshehri’s study^24^ that applied lipid concentration above 1 mM in general caused significant toxicity. Thus, all subsequent experiments were performed at that lipid concentration of 0.125 mM.

We further evaluated the cellular entry by using flow cytometry and fluorescent microscopy. As quantified in Fig. 4B, the maximal cellular uptake occurred with cationic DOTAP nanosystems (78.5% for DOTAP NLP and 79.8% for DOTAP NLG), as compared with neutral EPC nanosystems (61.7% for EPC NLP and 60.4% for EPC NLG). On the other hand, particle rigidity (NLPs versus NLGs) did not seem to affect the cellular entry. Fluorescent microscope images of cellular entry at 2 h are shown in Fig. 4C. NLGs were loaded with FITC-labeled siRNAs (green) in the chitosan hydrogel core and with the Rh-labeled lipid (red) in the lipid leaflets to evaluate differences in intracellular localization and to infer entry pathway. As seen in the fluorescent images of fibroGRO cells treated with both EPC and DOTAP NLGs, internalization was high and uniformly dispersed in the cytoplasm. Interestingly, for the DOTAP NLG system, the siRNA (FITC: green) and lipid (Rhodamine: red) were observed co-localized (in yellow) and widely distributed in the cytoplasm, suggesting that the cationic DOTAP nanolipogels could escape from endosomes after internalization and remain intact even after the endosomal escape for subsequent sustained siRNA release in the cytoplasm. In contrast, a wide separate distribution of green and red fluorescence dots was observed in the cytoplasm for the EPC NLG systemm.

### Endocytotic pathways of NLGs

**Figure 5:**
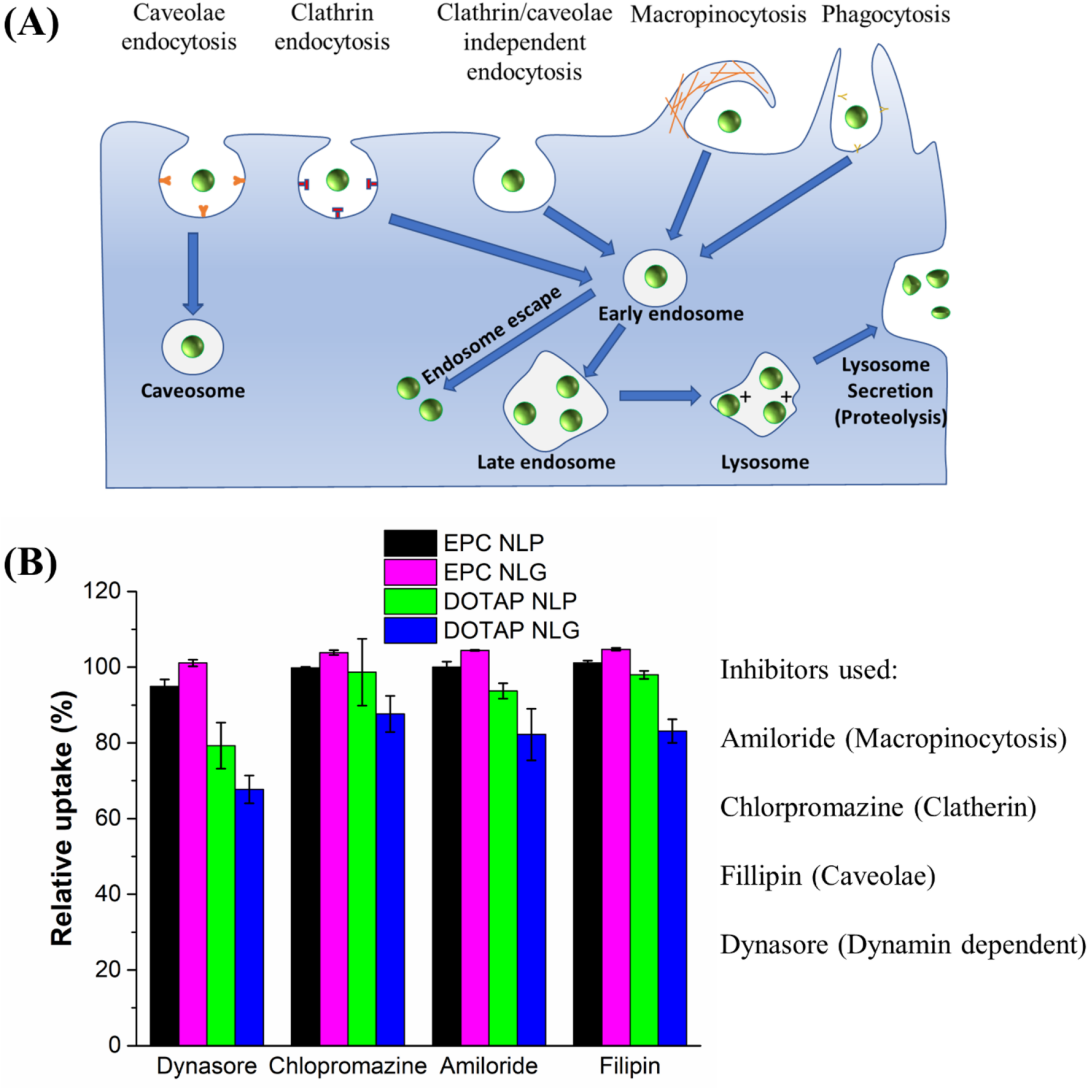
(**A**) NLGs mediates cellular uptake via different internalization pathways. (**B**) Relative cellular uptake of NLPs and NLGs in the presence of small molecules inhibiting macropinocytosis (Amiloride), clathrin (Chlorpromazine), caveolae (Filipin), and both endocytic pathways (Dynasore).

We used endocytosis inhibitors to assess the cellular internalization pathways (Fig. 5A) of the engineered NLP and NLG nanosystems. Four endocytosis inhibitors (Amiloride, Chlorpromazine, Filipin, and Dynasore) were used to inhibit macropinocytosis, clathrin-mediated, caveolae-mediated, and dynamin-mediated endocytosis, respectively. Amiloride is a commonly used inhibitor to block macropinocytosis by inhibiting Na^+^/H^+^ exchange at the cell surface^25^. Chlorpromazine is generally applied to redistribute the clathrin from the plasma membrane, making clathrin unavailable for assembly at the cell surface^20^. Filipin, a cholesterol-binding agent, inhibits the caveolae-mediated endocytosis pathway by binding to cholesterol inside caveolar pits and disrupting the pathway cycle. Lastly, Dynasore is a GTPase protein that selectively inhibits dynamin activity, which is essential for endocytic vesicle scission from cell plasma membrane during both clathrin- and caveolae-mediated pathways^25,26^.

Cellular entry of EPC based formulations was not affected by any of the endocytosis inhibitors (Fig. 5B). This could be due to both EPC NLPs and EPC NLGs entering cells predominantly via fusion, which is not affected by endocytosis inhibition. On the contrary, cellular entry of DOTAP NLP was significantly inhibited by Dynasore, indicating that the internalization of DOTAP NLP is prominently dependent on dynamin-mediated endocytosis. Interestingly, DOTAP NLG, by incorporating chitosan nanogel core with DOTAP lipid bilayer shell, exhibited a distinct internalization pattern from DOTAP NLP. In our investigation, all four inhibitors induced a significant inhibitory effect on the entry of DOTAP NLG at various extent. Dynasore still has the most inhibitory effect compared to the other pathway inhibitors Amiloride, Filipin, and Chlorpromazine. These results suggest that surface charge is a determinant factor in cellular processing of NPs and dynamin-mediated endocytosis is the predominant cell internalization pathway for cationic DOTAP nanosystems. Consequently, DOTAP NLG, unlike DOTAP NLP, adopts multiple endocytosis pathways, subsequently leading to superior gene silencing effect.

### Intracellular trafficking and gene silencing effect

**Figure 6:**
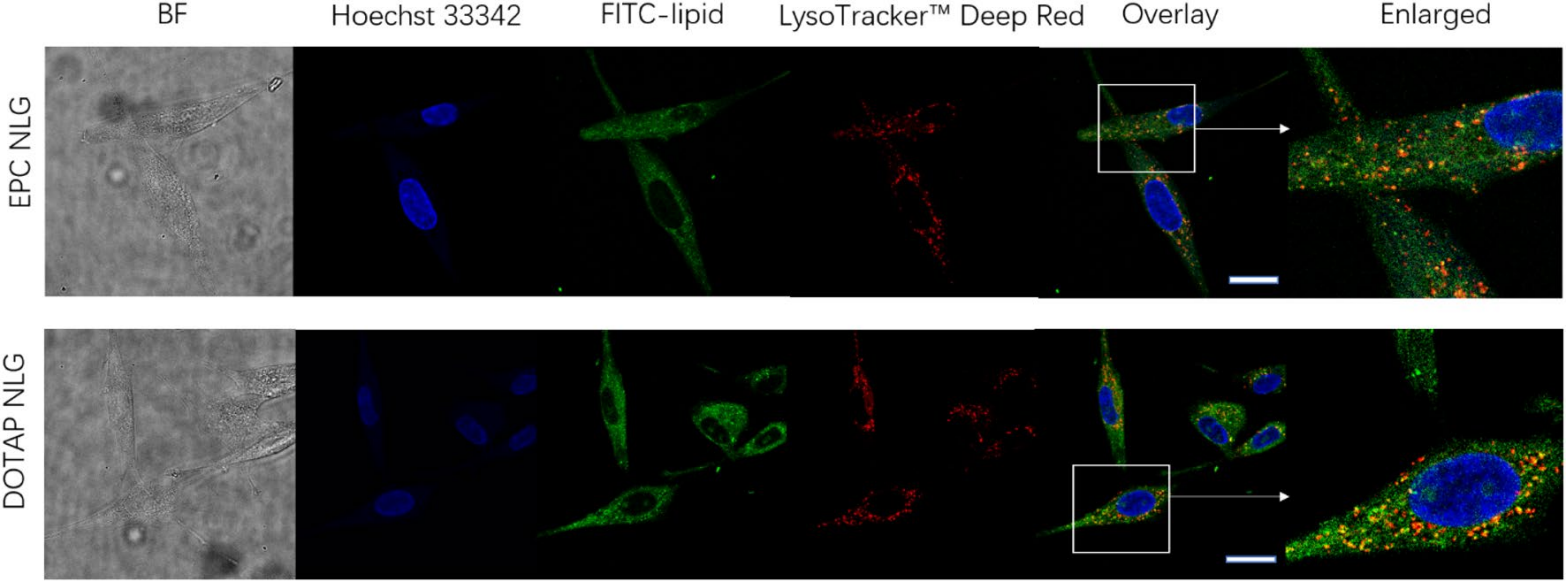
CLSM images of endolysosome trafficking of crosslinked EPC and DOTAP NLGs. (Blue: Hoechst 33,342, Green: FITC-lipid, Red: LysoTracker Deep Red, BF: bright field). The scale bar represents 25 µm.

To track NLGs following intracellular entry, fibroGRO were treated with FITC-labeled EPC and DOTAP NLGs and then stained with LysoTracker Deep Red. Intracellular localization was observed by CLSM in fibroGRO cells. As illustrated in Fig. 6, the endolysosomal compartments appeared as red dots and Hoechst-labeled nuclei exhibited blue fluorescence. Both EPC NLGs and DOTAP NLGs were observed intracellularly and co-localized with the lysosome (in yellow). A wide distribution of green fluorescence dots and lesser yellow dots could be seen in the cytoplasm in DOTAP NLGs, as compared with the EPC NLGs system. These results demonstrated that cationic lipid (DOTAP) could facilitate the endolysosome escape of DOTAP NLGs.

**Figure 7:**
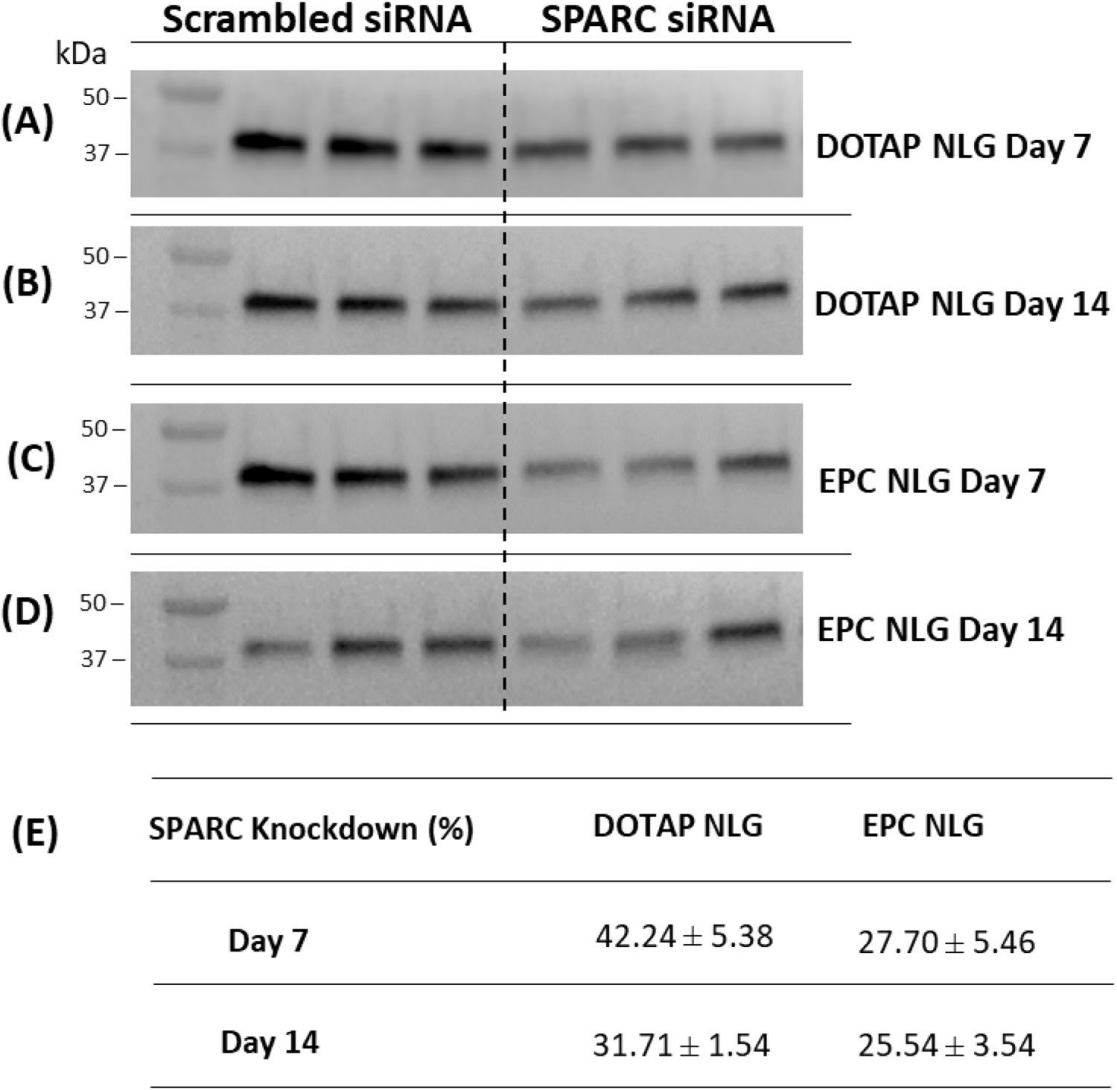
(**A**) DOTAP NLG DAY 7 SPARC Protein Band 1–3: Scrambled-siRNA, SPARC Protein Band 4–6: SPARC siRNA (**B**) DOTAP NLG Day 14 SPARC Protein Band 1–3: Scrambled-siRNA, SPARC Protein Band 4–6: SPARC siRNA (**C**) EPC NLG Day 7 SPARC Protein Band 1–3: Scrambled-siRNA, SPARC Protein Band 4–6: SPARC siRNA (**D**) EPC NLG Day 14 SPARC Protein Band 1–3: Scrambled-siRNA, SPARC Protein Band 4–6: SPARC siRNA. (**E**) Knockdown efficiency of crosslinked EPC and DOTAP NLGs encapsulated with SPARC-siRNA in fibroGRO cells (DOTAP NLG vs. EPC NLG, *p* < 0.05).

Secreted protein, acidic and rich in cysteine (SPARC) can modulate cell-extracellular matrix and elevated expression of SPARC have a strong association with the formation of tissue scarring and fibrosis^27^. SPARC targeted therapy has been evaluated as a potential therapeutic way to reduce SPARC production in human fibroblast cells and suppress the formation of fibrosis^28^. In this study, SPARC siRNA was encapsulated into EPC and DOTAP NLGs to evaluate the delivery and sustained-release effect in fibroblasts. Figure 7 depicted that the crosslinked NLG delivery system provides effective transfection and sustained effect in gene knockdown up to 14 days. At the protein level, the concentration of SPARC was consistently reduced in cells treated with SPARC siRNA. Crosslinked EPC NLG exhibits a reduction in SPARC transcription of 27.70% and 25.54% on Day 7 and Day 14, respectively. However, a higher knockdown was observed for DOTAP NLG on Day 7 (42.24%) and Day 14 (31.71%). Due to the absence of the nanogel network, uncrosslinked NLGs with encapsulated siRNA (Supplement information) have little to no significant knockdown as compared to their crosslinked counterparts.

## Discussion

Chitosan is an attractive and promising non-viral vector material for gene delivery, but none of the chitosan-based gene delivery carriers has been used clinically due to several challenges, such as poor water solubility, charge reduction at physiological pH and poor targeting capability. In this study, we proposed a simple one-pot method to fabricate core–shell CS NLG via liposome master templates for the controlled release of siRNA. The liposomal shell enveloping the chitosan nanoparticle core protects the chitosan-siRNA complex from dissociation at physiological pH and further facilitate the cellular internalization of NLGs. To start with, chitosan was the first methacrylate to form water-soluble and UV crosslinkable chitosan-methacrylate monomer. The synthesized CMA can readily form a complex with siRNA via electrostatic interaction and be further crosslinked to increase the structural stability of the nanostructure. The complex solution of CMA/siRNA was used to hydrate the lipid thin film so that the consequent self-assembly of lipids would encapsulate the complex. Extrusions of the multilamellar vesicles through a polycarbonate membrane filter with different sizes enabled them to achieve uniformed large unilamellar vesicles. After the removal of unencapsulated reagents by ultra-high centrifugation, the aqueous core of nanocarriers was UV-crosslinked to form a nanogel core/lipid shell structure. This novel liposome master template approach confers NLGs with homogeneous (PDI < 0.2) and controlled particle size.

^1^H-NMR results in Fig. 2A demonstrated the successful coupling between chitosan’s amino groups and methacrylic anhydride. The SD% of CMA was calculated from^1^H-NMR results and showed that 20% of primary amino groups were conjugated with acrylates. The unmodified 80% of primary amino groups have remained to complex siRNA. Particle DLS size (Table 1) confirmed this one-pot liposomal based fabrication approach can be used to fabricate homogenous nanoparticles for siRNA delivery. Zeta potential in Table 1 also demonstrated that siRNA was encapsulated inside the core of NLGs as EPC and DOTAP NLG showed neutral and positively charged, respectively. DLS images (Fig. 2B) and cryo-TEM (Fig. 2C) of NLGs confirmed the formation of hybrid core–shell nanostructure. To achieve sustained release of siRNA, we incorporated the CMA/siRNA complex and further crosslinked the CMA network into the interior of the liposome shells. Figure 3 presented that NLPs showed a high burst release (~ 60% on day 1) and finished the release at day 7. Contrary to the nanoliposomal system, the crosslinked NLGs provided superior siRNA entrapment (EPC NLG = 87% and DOTAP NLG = 79%) and were able to sustained-release of siRNA up to 28 days in vitro. Although EPC NLGs showed incomplete release of siRNA in vitro compared to DOTAP NLGs, intracellular release might be very different due to different endocytosis pathway and the escape of lysosome.

Surface charge is well known to be one of the key factors associated with endocytosis efficiency as well as the cytotoxicity of living cells^24^. As the cytomembrane possesses a negative charge, in this regard, cationic surface-charged nanoparticles may show a great extent of cellular entry^29^. Figure 4A showed the toxicity study with different amounts of NLG and no significant cytotoxicity was observed for both EPC and DOTAP NLGs when the lipid concentration was below 1 mM. In this study, neutral and positively charged NLGs was designed to evaluate the influence of surface charge on cellular entry and transfection efficiency. The neutral EPC NLG is composed of lipid bilayer consisting of EPC lipid, resulting in the zeta potential of 1.5 mV. Next, we modified the lipid formulation by incorporating a cationic DOTAP lipid (10%) in lipid bilayers of nanolipogels. This resulted in positively charged DOTAP NLG with a zeta potential of ~ 40 mV. In the cellular entry study, the results displayed that the internalization of cationic DOTAP nanosystems was enhanced as compared with neutral EPC nanosystems. Cellular binding is accelerated by electrostatic interaction between cationic DOTAP lipids and the cell membrane. Inevitably, the charge of the lipid bilayer plays a significant role in the initiation of cellular entry by modulating the interaction between the lipid bilayer of nanoparticles and the cell membrane.

Moreover, particle rigidity (NLPs vs. NLGs) did not affect the cellular entry. This result is contrary to the study by Guo et al., that have systematically investigated the role of nanoparticle rigidity in cellular entry using alginate-based nanolipogels and reported that cells exhibited significantly greater entry of soft NLGs relative to their elastic counterparts^30^. This is potential because the chitosan hydrogel core in NLG is weakly crosslinked in our current studies, and at such low elasticity, the elasticity-meditated cellular uptake effect is negligible. Therefore, it suggests that surface charge plays an important role in facilitating cellular entry in this study. Moreover, the difference in the CLSM images (Fig. 4C) between DOTAP and EPC NLGs provides indirect evidence that cationic DOTAP NLGs could escape from endosomes after cellular internalization and remain intact for subsequent siRNA sustained release in the cytoplasm. These results lead us to hypothesize that the cell internalization pattern could be noticeably different between DOTAP NLG and EPC NLG.

To validate this hypothesis, endocytosis inhibition of different nanosystems in the presence of Dynasore, Chlorpromazine, Amiloride, and Filipin was investigated. Cellular entry of EPC based formulations was not affected by any of the endocytosis inhibitors, showing no clear preference for specific routes and predominantly entering cells via fusion. In contrast, cellular entry of DOTAP NLP was significantly inhibited by Dynasore, indicating that the internalization of DOTAP NLP is prominently dependent on dynamin-mediated endocytosis. Furthermore, the intracellular entry of DOTAP NLG was found to be significantly reduced in the presence of all four inhibitors at various extent, indicating that besides dynamin-mediated endocytosis, multiple pathways were involved in the endocytosis process. Taken together, these results suggest that surface charge is a determinant factor in cellular processing of nanoparticles and dynamin-mediated endocytosis is the predominant cell internalization pathway for cationic DOTAP nanosystems.

Although there were no significant differences in the extent of cellular entry among the DOTAP nanosystems, the mechanism of entry differs among these systems. In particular, DOTAP NLG which can enter the cells via multiple pathways including caveolae-mediated endocytosis could subsequently offer superior gene silencing effect by partly bypassing lysosomes to avoid lysosomal degradation. Furthermore, the cationic DOTAP NLG internalized by the clathrin-medicated pathway could effectively escape endosomes via the proton sponge effect (the interactions of cationic lipids with the anionic components of the endosomal membrane). After the endosomal escape, these covalently crosslinked chitosan NLGs can release siRNA moderately in the cytoplasm, overcoming the drawbacks (poor stability and lack of sustained-release) of the current chitosan complex nanoparticles. Endolysosome trafficking images in Fig. 6 illustrated that there was more green fluorescence (NLG alone) in the cytoplasm and less co-localization (NLGs inside endo/lysosome) for DOTAP NLGs compared to EPC NLGs. Those results indicated that cationic DOTAP NLG has a better capability for endolysosome escape.

Following the successful entry of the siRNA carriers into the targeted cells and escape from endolysosome, the release of siRNA within the cells is equally important for sustained gene knockdown. To evaluate the transfection efficiency over time, western blot experiments were conducted to evaluate the SPARC expression in fibroblasts treated with various NLG nanosystems. The results revealed that DOTAP NLG exhibited significantly higher in-vitro transfection efficiency than the EPC NLG system. This could be attributed to the higher cellular entry, multiple endocytosis pathways and improved capability for endolysosome escape of the cationic DOTAP NLG. While fibroblast cells have a doubling time of 2–3 days^31^, the low expression of SPARC protein at day 14 in Fig. 7 further verified the sustained release of siRNA from NLGs induced long-term gene knockdown. This is attributed to the diffusional control exerted by the crosslinked core to the escape of either complexed or uncomplexed siRNA. The importance of having crosslinked NLG core was confirmed by the fact that the uncrosslinked DOTAP NLG system has little to no knockdown. It may be due to the decomplexation of the CMA/siRNA complexes in the uncrosslinked core at pH7.4 and the lack of diffusional control offered by crosslinking the core. This observation agrees with previous studies, which showed that nanoparticles formed via siRNA complexation (but no crosslinking) typically only have efficient gene knockdown in the early 1–2 days^20,32^. Taken together, our results have demonstrated the novelty of the non-viral crosslinked NLG delivery system that offers a safe and efficient delivery vehicle for the prolonged gene silencing.

## Conclusions

In summary, this work introduced a new long-acting siRNA delivery platform based on siRNA/CMA complex nanolipogels via the liposome template. The complex of siRNA/CMA enhanced the encapsulation efficiency of siRNA and retained the activity of siRNA. The complex was then incorporated into liposomes and then the chitosan was crosslinked to form nanolipogels. Our experiments proved that crosslinked NLG provided sustained release of siRNA for 28 days and prolonged gene silencing up to 14 days compared to the uncrosslinked NLG. Therefore, UV crosslinking of CMA nanogel inside NLG offered the NLG with the ability of sustained-release siRNA and prolonged gene silencing. Additionally, NLGs provide the advantage of being prepared with a high loading of siRNA compared to solid lipid nanoparticles. NLG is a promising sustained-release system to prolong siRNA delivery and control gene expression over time. We expect that our approach can be extended to targeted siRNA delivery for specific types of cells or tissues.

## Methods

### Materials

Chitosan, (deacetylated degree: 85%, average MW: 15,000) were obtained from Polyscience Inc. Methacrylic anhydride, Irgacure 2959(I2959), acetic acid and deuterium oxide were obtained from Sigma Aldrich. 6-Carboxyfluorescein (6FAM) SPARC siRNA and normal SPARC siRNA with Mw of 13,369 g/mol were achieved from Shanghai Shenggong Corp. The sense sequence of SPARC siRNA is AACAAGACCUUCGACUCUUCC, while the antisense sequences is GGAAGAGUCGAAGGUCUUGUU. Both of them were unmodified. The negative control scramble siRNA was purchased from Shanghai Shenggong Corp. 95% Egg PC(EPC, from egg chicken) and 99% 1,2-dioleoyl-3-trimethylammonium-propane (chloride salt) (DOTAP) were obtained from Avanti Polar Inc. Quant-iT™ RiboGreen™ RNA Assay Kit, DMEM medium and fetal bovine serum were purchased from Thermo Fisher Scientific. FibroGRO cells were achieved from ATCC Cell lines company.

### Chitosan-MA (CMA) synthesis

A 3 wt. % of chitosan solution was prepared with 2% acetic acid at room temperature with constant stirring overnight. Methacrylate anhydride was added dropwise at 1 molar equivalents per chitosan repeating unit. The mixture reacted at 60 °C for 6 h and then was dialyzed (molecular weight cut off 3 K, Thermo fisher) against distilled water for 4 days with 3 changes of distilled water each day^33^. The purified CMA was then lyophilized and stored in − 20 °C until use. The degree of methacrylic modification of chitosan was determined using ^1^H NMR spectroscopy and recorded on a Bruker NMR (ADVANCE III, 400 MHz) with D_2_O as the solvent. The ^1^H NMR spectroscopy of unmodified chitosan was presented in the SI. Size exclusion chromatography (SEC) using PL-Aquagel OH Mixed-H column (8 µm) was used to analyze the molecular weight of CMA according to the Application note of Agilent SEC Analysis of Chitosan. The experiment was performed in Agilent 1260 Infinity SEC system with RI detector. The mobile phase was 0.5 M NaNO_3_ + 0.01 M NaH_2_PO_4_ at pH 2 with the flow rate of 1.0 mL/min. The substitution degree (SD) of CMA was determined from the ratio of the integrated area of the Hb- Hf peaks to that of the methylene (H_g_) peaks according to the Eq. (1)^33^.

1 $$SD = \frac{{\frac{Area\,of\; Peak\; g}{2}}}{{\frac{Area \;of\; Peak\; b, c, d, e, f }{5}}} \times 85\%$$

### NLG and bare liposome fabrication

60 mg EPC was dissolved in 3 mL chloroform and EPC thin film was prepared by a rotary evaporator rotating at 150 rpm for an hour at 40 °C. 30 mg CMA was dissolved in 6 mL 0.9% NaCl solution with 0.01 mol/L HCl to obtain a homogenous mixture, while 2.5 mg/mL photo initiator I2959 and 30 nmol SPARC siRNA were added to dissolved CMA solution. This solution was used to hydrate the lipid thin film for an hour. After hydration, extrusion was conducted through 0.4 µm (3 times) and 0.2 µm (5 times) Polycarbonate membranes (Whatman GE) to downsize the multilamellar vesicles to unilamellar vesicles. Uncrosslinked NLG pellet was achieved by the use of ultra-high centrifuge (300,000 g, 1 h) and then re-suspended in 8 mL pH7.4 PBS. Finally, crosslinked NLG was polymerized from above purified 4 mL NLG solution under UV (VL-8. L, Vilber, 1-2 mW/cm^2^) at 365 nm for 30 min, while the remaining solution was the uncrosslinked NLG solution. DOTAP NLG was prepared by the same method except for the use of 54 mg EPC (90%) with 5.5 mg DOTAP (10%) during thin film preparation. Bare EPC and DOTAP liposomes were fabricated using the same protocol without the use of CMA during the hydration step.

### Encapsulation efficiency of siRNA and release study

The release of SPARC siRNA from liposome to NLG was measured over a stipulated period of 28 days. 1 ml of samples was added to a dialysis bag (100kD, MWCO, Spectrum) immersed in 14 ml PBS, and put in a thermo shaker (100 rpm, 37 °C). RiboGreen Assay was used to quantify the release amount of siRNA in the release buffer. For each time point, the release buffer was fully exchanged with fresh PBS. The data obtained were compared against a standard curve generated from a series of known concentrations of SPARC siRNA.

Triton X-100 will decrease the sensitivity of the Ribogreen assay, so the encapsulation efficiency of siRNA is calculated from the sum of the cumulative release amount and the remaining amount in Uncrosslinked NLG after day 28. To determine the remaining amount of siRNA in Uncrosslinked NLG, Uncrosslinked NLG was mixed with 1% Triton X-100 (v/v). The siRNA concentration was determined by the RiboGreen assay, while the standard curve was tested with the same amount of Triton X-100.

### Characterization studies

After encapsulation of siRNA (30 nmol) into NLGs and liposomes, the size and zeta potential of the nanoparticles were evaluated using Malvern Zetasizer 2000. The sample was diluted 100 × in DI water before dynamic light scattering (DLS) measurement. To prove the nanogel-core structure, 1% Triton-X100 was used to strip off the lipid bilayer and then diluted 100 × prior to DLS measurement.

The morphology of the liposome and NLGs were confirmed by Cryo-Transmission Electron microscopy (Cryo-TEM, Carl Zeiss AG-Libra 120 Plus TEM microscope, Germany) via the plunge freezing method. The samples were prepared by dripping 3µL of sample onto the 300-mesh lacey carbon grid, blotted and plunged into the liquid ethane. The samples were soaked in liquid nitrogen and then imaged by Cryo-TEM at 120 kV with Gatan 6226 cryo-holder. Subsequently, the sample was imaged with a 2 k × 2 k CCD camera while the experiment was maintained below -170 °C all the time.

In this study, lipid concentration in the nanoparticles was determined by modified Stewart Assay through quantifying the phospholipids complex form with ammonium ferrothiocyanate^34^.

### Cell toxicity study

Human foreskin fibroblast (FibroGRO) cells were purchased from Sigma Aldrich, maintained in DMEM media with 10% FBS and cultured in a CO_2_ incubator with 5% CO_2_ at 37 °C. Cells at passage 5 or less were used in this study. Cell toxicity study was conducted using MTT Assay to determine the cytotoxicity of the NLGs. 500 FibroGRO cells were seeded in the 96 well plates (Thermo Fisher), and administered with increasing concentration of the NLGs encapsulated with SPARC siRNA after seeding the cells for 12 h. After the treatment of 1 day, a solution of 10% of 12 mM Thiazolyl Blue Tetrazolium Bromide solution mix in PBS was used to dye the cells. DMSO was added to each well after 4 h of incubation. After 10 min of shaking, Tecan Microplate reader is used to measure absorbance at 540 nm. The cell viability can be calculated from the absorbance, while the viability of untreated cells was assumed as 100%.

### Cellular entry studies

Cellular entry of nanoparticles was appraised by confocal microscopy and flow cytometry. A qualitative study involves the usage of the CLSM 510 Confocal Microscope (Leica, Germany). FibroGRO cells were seeded in 4 wells coverslip base chambers (Nalgene, Nunc International, Naperville, IL, USA) at 5000 cells per well, treated with NLG containing 0.0625 mM lipids and FITC-SPARC siRNA and incubated 2 h at 37 °C. Before doing the imaging, FibroGRO cells were treated with Hoechst33342 (2 µg/mL, Thermo Fisher) and washed twice by PBS. All sample images were compared with untreated cells with the same imaging setting to verify the controls showed no green FITC signals and red Rhodamine signals.

Quantitative cell entry was performed via flow cytometry (Guava® easyCyte 8HT Benchtop Flow Cytometer, Merck-Millipore). In this study, 100,000 FibroGRO cells were treated with NLG containing 0.0625 mM lipids with 0.1 mol % (1,2-dioleoyl-sn-glycerol-3-phosphoethanolamine-N-(carboxyfluorescein) (ammonium salt), Avanti) and then incubated 2 h at 37 °C. After trypsinization and washing, the cells were fixed at 4% paraformaldehyde (PFA) in PBS for 20 min at RT and then washed with PBS. The fluorescence inside cells was determined via flow cytometry and data was analyzed by GuavaExpress Pro software (EMD Millipore). The treated samples were normalized by the untreated cells (FibroGRO only).

### Endocytosis inhibition and endolysosome trafficking

To elucidate potential cellular entry pathways of NLGs and NLPs, 100,000 FibroGRO were pre-treated with selective chemical endocytosis inhibitors at a concentration of 100 µM Amiloride, 2 µM filipin III, 10 µM chlorpromazine, and 100 µM dynasore. After treating with inhibitors for 30 min, freshly prepared FITC-lipid incorporated NLGs and NLPs were added to the cells and incubated for 1.5 h. After trypsinization and washing, the cells were fixed at 4% PFA in PBS for 20 min at RT and then washed with PBS. The fluorescence inside cells was determined via flow cytometry according to the methods described above. The different nanoparticles without any inhibitor treatment were used as control groups. The fluorescence intensity of each group treated with inhibitors was normalized by the intensity of the control groups.

To study the intracellular trafficking of EPC and DOTAP NLG, 5000 FibroGRO were seeded in 4 well chamber and cultured overnight. Cells were treated with FITC-lipid incorporated EPC and DOTAP NLGs for 1.5 h. Subsequently, cells were washed with PBS and then incubated LysoTracker™ Deep Red (Thermo Fisher, 50 µM) for 30 min. After the incubation, the cells were fixed by PFA and stained with Hoechst 33,342 according to the procedure described in section 2.7 and finally imaged with CLSM.

### Western blot experiments

FibroGRO cells (50,000) were seeded on a 6-well plate with 2 mL DMEM media and cultured for 24 h. After changing the medium to serum-free DMEM, SPARC siRNA and scrambled siRNA of crosslinked and Uncrosslinked EPC and DOTAP NLGs were treated to the cells. The same amount of siRNA (0.25 nmol siRNA per 50,000 cells) equivalent nanoparticles for the various sample groups were treated to the cells for fair comparisons. Fresh DMEM with serum was added after 12 h incubation with the nanoparticles. Subsequently, the medium was added every three days and cells were harvested, lysed after 7 and 14 days of culture. Extraction of protein was harvested using a lysis buffer at 7 and 14 days, the extracted protein was analyzed by using Coomassie blue (Bio-Rad) at 595 nm. Comparable amount (30ug) of protein samples and ladder (Bio-Rad, precision plusTM) are loaded into the gel (Bio-Rad, MP SFX, 4–20% 10WDEE) for protein separation. Chemidoc imaging system (Bio-Rad) was used to validate the protein separation step, moreover, the protein transfer to the PVDF membrane was performed via a dry (Bio-Rad) system. The membranes' image was captured to validate the protein transfer, further the membranes were blocked in 5% skim milk for 1 h at room temperature. Antibody treatment was performed by membranes probing with primary antibody (mouse monoclonal IgG1, Santa Cruz), which was diluted 1000 times in 2.5% skim milk and subsequently incubated for 1 h at RT. After washing the membrane with TBST for three-time (10 min interval), horseradish peroxidase linked anti-mouse secondary antibody (Jackson Immuno Labs) with the dilution of 1:10,000 was incubated at RT for 1 h and washed using TBST for six times intermittently at 5 min. Bands were digitally exposed using chemiluminescent substrate and image using Image Lab software (Bio-Rad). For relative quantification, GADPH was used as the housekeeping protein. For relative quantification of SPARC protein levels, the band corresponding to SPARC at 43 kD was selected with triplicate runs and mean value with standard deviation is reported.

### Statistical analysis

Statistical significance was determined by a two-sample student’s t-test. A *p* < 0.05 is considered statistically significant (*p < 0.05).

## Supplementary Information

Supplementary Information.
