# Supplementary material for: Designing siRNA/chitosan-methacrylate complex nanolipogel for prolonged gene silencing effects
Source: Sci Rep. 2022 Mar 3;12:3527. doi: 10.1038/s41598-022-07554-0 (PMC8894398; doi:10.1038/s41598-022-07554-0)
Supplement: Supplementary file 1 — Supplementary Information. [file 41598_2022_7554_MOESM1_ESM.docx]

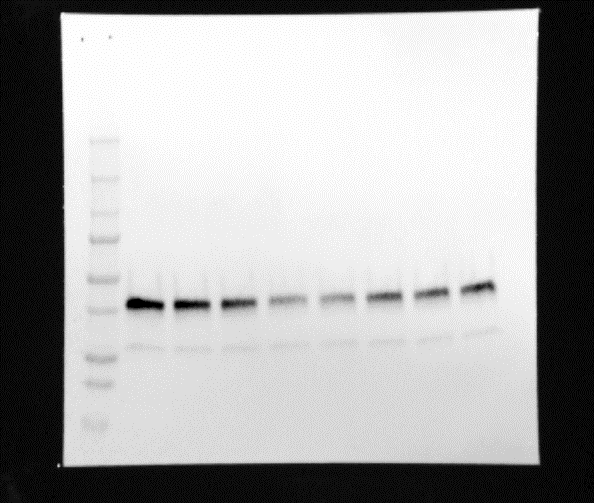

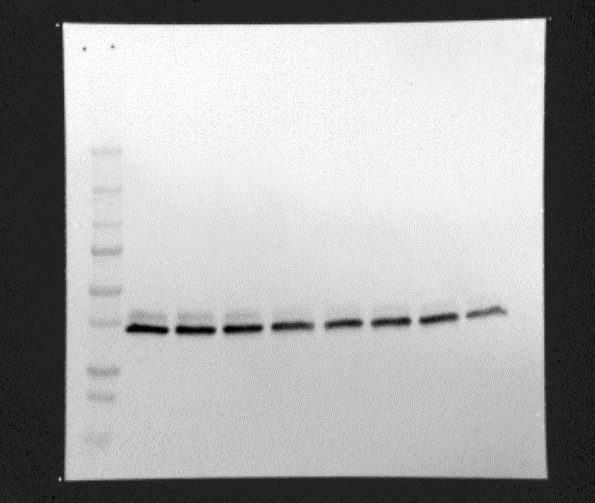


**(B)**

**(A)**

**Figure S1：Day 7 EPC** FibroGRO cell samples post 7 days after treatment with EPC NLG NP encapsulated with scrambled/SPARC siRNA. (A) SPARC Protein Band 1-3: Scrambled-siRNA, SPARC Protein Band 4-8: SPARC siRNA (B) GAPDH Protein Band 1-3: Scrambled-siRNA, GAPDH Protein Band 1-3: SPARC siRNA.

**
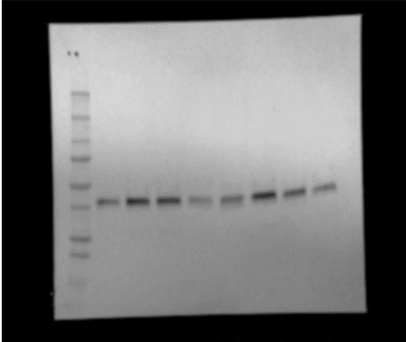

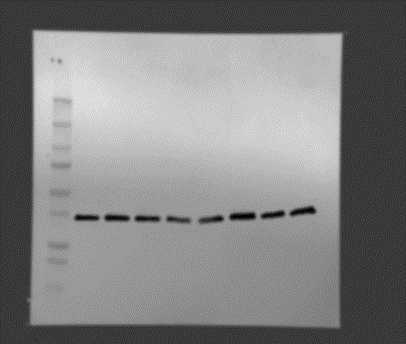
**

**(D)**

**(C)**

**Figure S2: Day 14 EPC** FibroGRO cell samples post 14 days after treatment with EPC NLG NP encapsulated with scrambled/SPARC siRNA. (C) SPARC Protein Band 1-3: Scrambled-siRNA, SPARC Protein Band 4-8: SPARC siRNA (D) GAPDH Protein Band 1-3: Scrambled-siRNA, GAPDH Protein Band 1-3: SPARC siRNA.

**(F)**

**(E)**


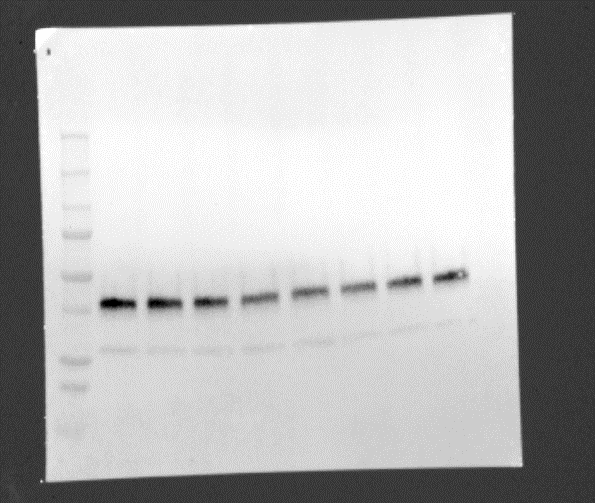

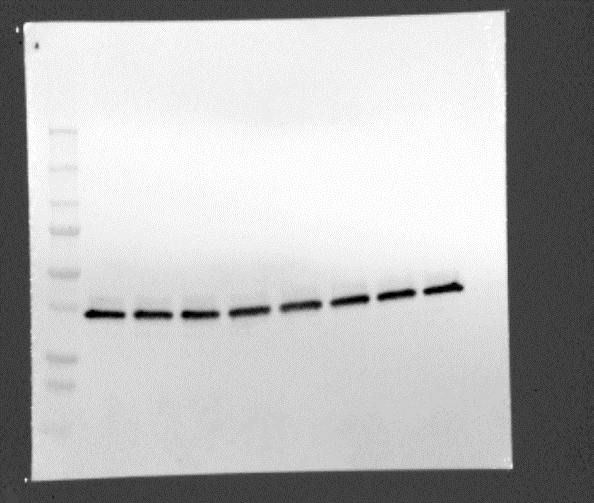


**Figure S3:Day 7 DOTAP** FibroGRO cell samples post 7 days after treatment with DOTAP NLG NP encapsulated with scrambled/SPARC siRNA. (E)SPARC Protein Band 1-3: Scrambled-siRNA, SPARC Protein Band 4-8: SPARC siRNA (F) GAPDH Protein Band 1-3: Scrambled-siRNA, GAPDH Protein Band 1-3: SPARC siRNA.


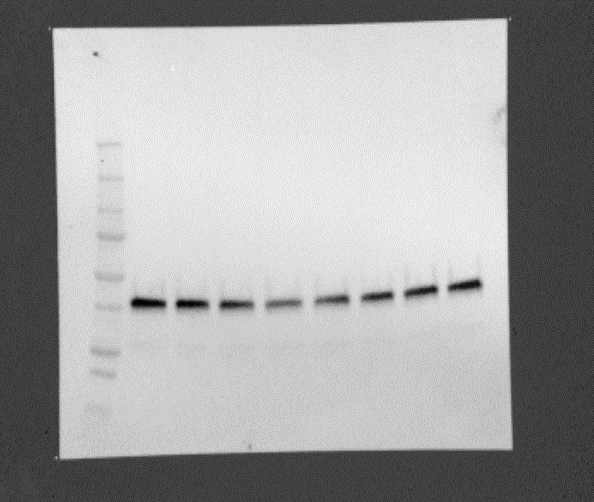

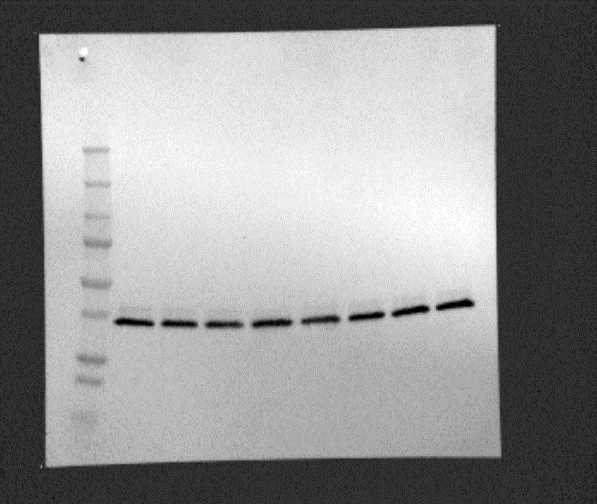


**(G)**

**(H)**

**Figure S4: Day 14 DOTAP** FibroGRO cell samples post 14 days after treatment with DOTAP NLG NP encapsulated with scrambled/SPARC siRNA. (G)SPARC Protein Band 1-3: Scrambled-siRNA, SPARC Protein Band 4-8: SPARC siRNA (H) GAPDH Protein Band 1-3: Scrambled-siRNA, GAPDH Protein Band 1-3: SPARC siRNA.
